# Stress and Depression: Preclinical Research and Clinical Implications

**DOI:** 10.1371/journal.pone.0004265

**Published:** 2009-01-30

**Authors:** Alessandro Bartolomucci, Rosario Leopardi

**Affiliations:** 1 Department of Evolutionary and Functional Biology, University of Parma, Parma, Italy; 2 Department of Clinical Neuroscience, Karolinska Institutet, Stockholm, Sweden; James Cook University, Australia

Major depression (MD) is a severe, life-threatening, and highly prevalent psychiatric disorder, predicted to soon become one of the major causes of death worldwide. Despite extensive investigations, the exact mechanisms responsible for MD have not been identified. This Overview focuses on the role of stress in depression. It discusses current advancements in biological psychiatry, neuroscience, and neuroendocrinology, highlighting key findings presented in the research papers included in the *Special Collection* “Stress-Induced Depression and Comorbidities: From Bench to Bedside,” published in this issue of *PLoS ONE*. The Overview encompasses the problematic diagnosis of MD, as well as preclinical evidences linking genetic predisposition, early life events and social factors, imbalanced HPA axis, molecular pathways within the central nervous system, and metabolic comorbidities with depression-like disorders. It is emphasized how the link between stress and depression can be deeper than previously recognized, following the description of a potentially common depression subtype, named tentatively “stress-induced depression” (STRID). Due to the inherent biological perspective underlying the STRID concept, both preclinical and clinical research will be pivotal in clarifying the validity of this new subtype of MD and in improving predictors for treatment response, and will provide a better basis for genetic studies as well as stimulating new drug discovery programs.

## Introduction

Major depression (MD) is a severe, life-threatening, and widespread psychiatric disorder having an incidence of about 340 million cases worldwide. MD ranks fifth among leading causes of global disease burden including developing countries, and by year 2030 it is predicted to represent one of the three leading causes of burden of disease worldwide [1], [2]. MD is also a risk factor for cardiovascular and metabolic diseases, and a major risk factor for suicide [3]. Despite extensive investigations, the exact mechanisms responsible for MD have not been identified, and current therapeutics are based on serendipitous discoveries rather than on bench-to-bedside, targeted drug discovery [4]. In addition, although clinically efficient antidepressant drugs do exist, the situation is in many cases far from ideal. Shortcomings such as low remission and/or high treatment-resistance rates, slow onset of action, side effects, and drug–drug interactions merit the exploration of all plausible agents that are effective, tolerable, and safe, and that improve maintenance of wellness [5]–[8]. Accordingly, there is an enormous need for joint experimental efforts between preclinical and clinical scientists.

Understanding MD in its etiology and biological phenomenological characteristics could improve its recognition and treatment [8]–[11]. The present Overview highlights current trends in modern biological psychiatry, neuroscience, and neuroendocrinology by discussing key aspects presented in research papers included in the *Special Collection* “Stress-Induced Depression and Comorbidities: From Bench to Bedside,” published in this issue of *PLoS ONE*.

## The Problematic Diagnosis of Major Depression

Presently accepted diagnostic criteria for MD [12] are five (or more) specific symptoms having been present during the same two-week period and representing a change from previous functioning; at least one of the symptoms should be either depressed mood or loss of interest or pleasure. Although their definition followed rigorous statistical validating criteria and years-long investigations, they are often criticized as being subjective–qualitative rather than objective–quantitative [13], [14]. The current *Diagnostic and Statistical Manual* (*DSM-IV*) classification ignores etiology, and distinguishes between bipolar and unipolar conditions, and within the unipolar group between cases with and without melancholia, or with and without psychotic symptoms, as well as atypical depression. In addition, the current diagnostic criteria represent clusters of symptoms and characteristics of clinical courses that do not necessarily describe homogenous disorders and may rather reflect common final pathways of different pathological processes [15]. MD is also a highly heterogeneous disease. Subtypes of depression may differ not only in etiology and clinical picture, but also in clinical response to medical treatments [15]. However, no past or present classification includes biological criteria except for changes in body weight or sleep parameters [16]. Therefore, there is an urgent need for neurological, biological, and genetic data in future *DSM* classifications [17]. Inclusion of biological diagnostic criteria requires extensive investigation on the biological correlates of MD as well as on the implementation of mechanistic-based investigations. In this respect, Åsberg and coworkers [18] and Schüle and coworkers [19] add to the current discussion important biological correlates in MD patients. Increased plasma monocyte chemoattractant protein-1 (MCP-1), epidermal growth factor (EGF), and vascular endothelial growth factor (VEGF) are increased in a population of women under prolonged psychosocial stress and can thus be considered potential biomarkers for screening and early interventions [18]. These data are particularly intriguing because they extend a growing body of evidence linking increased plasma concentration of signaling molecules such as cytokines and growth factors with MD [13], [20], [21]. On the other hand, hypothalamo–pituitary–adrenocortical (HPA)-axis dysregulation is confirmed to be an important parameter for treatment outcome in MD by Schüle and coworkers [19], although dysfunction of the HPA system as determined by the classical dexamethasone (DEX)/corticotrophin releasing hormone (CRH) test as well as with other neuroendocrine assays, seems to be neither a necessary nor a sufficient determinant for acute treatment response. Of interest from a biological perspective, Czéh and coworkers [22] recently observed that chronic tianeptine treatment may reverse neurobiological alterations associated with chronic psychosocial stress in male tree shrews without an improvement in HPA functions (see also [23]).

## Genetic Predisposition

Although no single gene could be responsible for a complex and multifactorial disorder like MD, association and pharmacogenetic studies identified a number of loci associated with vulnerability to MD or antidepressant efficacy [8], [10], [24]–[26]. In parallel, increasing evidences report gene×environment (G×E) effect in MD, with stress often representing the key environmental trigger of MD onset in vulnerable individuals [27]. Preclinical studies are ongoing to elucidate specific genes and environmental context that could precipitate psychopathologies [28]. An alternative approach by Touma and coworkers [29] is described here: They selected for seven generations of mice based on their corticosterone reactivity to an environmental challenge. They demonstrate that individuals selected to be high responders show blunted circadian rhythmicity of HPA-axis hormones, behavioral hyperactivity, and changes in rapid eye movement (REM) and non-REM sleep, as well as slow wave activity, indicative of reduced sleep efficacy. Considering the incidence of disturbed HPA axis and sleep disorders in MD patients, these selected mouse lines may offer a new important experimental tool.

G×E effects are easily accessible for preclinical investigations, and the elevated number of inbred strains of mice (i.e., animals showing almost null heterozygosis) available offers a powerful experimental tool. In this respect, Schweizer and coworkers [30] further clarify how different inbred strains of mice can have very different vulnerabilities to the chronic mild stress model of depression in both behavioral and physiological parameters, thus offering an invaluable tool to understanding G×E interaction in stress-induced disturbances.

## A Biological Pathogenesis: The Stress Model and HPA Axis Activity

Stress is usually defined as a state of disturbed homeostasis inducing somatic and mental adaptive reactions, globally defined as “stress response,” aiming to reconstitute the initial homeostasis or a new level of homeostasis after successful adaptation, i.e., allostasis [31]–[34]. There is wide consensus and support from preclinical and clinical data that stress exposure conceivably plays a causal role in the etiology of MD and depression-like disorders [11], [27], [31], [34]. However, no specific mechanism linking stress exposure and stress response to the occurrence of MD has yet been fully elucidated. Growing evidence indicates several classical candidates, including neurotransmitters and neuropeptides, as well as conceptually novel immune and inflammatory mediators, as likely intermediate links between stress exposure, depressive symptoms, and MD [9], [21], [34]–[38]. Related to the latter, Åsberg and coworkers [18] discuss in their paper in this *Collection* a potential role for some inflammatory mediators in a cohort of patients under prolonged psychosocial stress, providing further epidemiological support (results discussed above in this Overview).

One of the hallmarks of the stress response has long been considered the activation of the HPA axis. Hypothalamic CRH activation is a pivotal signaling molecule in the regulation of the HPA axis in particular and of the stress response in general. Therefore, comprehension of the mechanism responsible for the negative feedback regulation of CRH is of paramount importance. In the present *Collection*, van der Laan and coworkers [39] demonstrate that the timing of glucocorticoid receptors (GR) activation determines the effective repression of the cAMP-induced transcription of the CRH gene, thus clarifying that *in vivo* a critical time window may exist for effective repression of the CRH gene and HPA axis by glucocorticoids.

Knowledge on the functioning of the HPA axis under acute or chronic challenge is also a key to understanding the intimate link between stress response and the pathogenesis of depression [40]. Indeed, in all MD syndromes, a certain degree of HPA-axis disturbance is often present, visible either at the baseline or with functional tests. Despite the fact that observed changes of HPA regulation are so far not specific for the diagnosis of depression or for any of its clinical syndromes [8], altered HPA-axis parameters are considered important biomarkers, particularly in preclinical studies. Increased circulating hormones such as adrenocorticotropic hormone (ACTH) and cortisol/corticosterone or increased adrenal gland weight are considered biomarkers of stress response in preclinical models [41], including in several papers in this *Collection* [19], [29], [42]–[46]. Despite the bulk of data available, surprisingly current knowledge has not yet been developed to a point where HPA-axis reactivity can be rationally exploited for targeted drug treatment, as opposed to the major achievements of drugs targeting the CRH receptors [47]. Present data offer reliable experimental tools to stimulate future drug discovery programs [48].

## Behavioral Neuroscience

The *DSM-IV* identifies specific behavioral and cognitive diagnostic criteria for MD patients. Among these, depressed mood, anhedonia, locomotor disturbances, and anxiety are accessible for preclinical investigation, while others such as feelings of worthlessness and thoughts of death or suicide cannot be reliably mimicked in animal models. A number of animal models have been developed and validated [11], [38], [49]–[51]. In particular, models involving a chronic (i.e., continuous exposure to a threatening stimulus for a significant amount of time, usually weeks) or intermittent (i.e., daily short exposure to a threat for subsequent days) exposure to negative stressful events can be considered the most effective in modeling MD-associated behavioral and physiological disturbances (but see [52]). In line with this conclusion, the preclinical papers included in the *Special Collection* of *PLoS ONE* make use of chronic or intermittent models of stress [30], [42]–[45]. Furthermore, papers in the *Collection* also describe animal models that are increasingly being regarded as the most promising to model etiological factors and key features of MD patients, i.e., i) models in which the threatening stimulus is social in nature [42], [46], ii) models in which exposure to stressful stimuli occurs in the early postnatal or juvenile age [44], [46].

From a nosological point of view, original research presented in the *Collection* further clarifies that at least some behavioral disturbances present in MD diagnostic criteria can be reliably induced and experimentally determined in animals models, including for anhedonia [30], locomotor disturbances [29], [42], [44], [53], anxiety [44], [53], sleep pattern [29], and learning/memory [44], [53]. Of main interest is the experimental evidence [30] that a shift in the circadian rhythm induced by overnight illumination is the single most important experimental factor influencing the intake of a sweetened solution, which is the currently accepted animal equivalent of evidence of anhedonia [54]. Furthermore, Ilin and Richter-Levine [44] show that daily exposure to a stressful stimulus for three consecutive days at a juvenile age (named juvenile stress, JS), determines long-lasting behavioral and motivational effects in rats, i.e., increased anxiety, lower exploratory drive, and increased learned helplessness. On the contrary, housing JS rats in an enriched environment completely abolished JS-induced behavioral effects.

A similar ameliorating effect on stress coping has also been determined by Collins and coworkers [53], by exposing rats to a different form of environmental enrichment, i.e., home cage presence of a running wheel which induces in rodents spontaneous physical exercise. Exercised rats show increased behavioral coping and reduced anxiety and depression-like behaviors in the open field test and in the forced swimming test when compared with sedentary rats.

## Molecular Neuroscience

In the postgenomic era, high-throughput techniques allow the identification of genes overexpressed or downregulated in selected brain regions after chronic stress exposure or in MD. Following these observations, researchers now aim at translating *omics* evidences into experimentally based results (based on *on purpose* experimental designs). For example, the “sequenced treatment alternatives to relieve depression” (STAR*D) clinical trial identified several loci associated with response to anti-depressants and suicidal ideation in MD patients [55], [56]. Among these is the gene encoding for a class of ionotropic glutamate receptors known as kainate receptors (KA). Hunter and coworkers [45] now report that KA1 subunit mRNA is selectively modulated by stress- and HPA-axis activity in the dentate gyrus and CA3 region of the hippocampal formation. Another fruitful approach follows the identification of potential biomarkers in postmortem brain tissue of MD patients. A recent study found in the prefrontal and parieto-occipital cortex of MD patients an altered level of the L1-cell adhesion molecule (L1-CAM) [57]. Ilin and Richter-Levin [44] firstly demonstrate that juvenile stress in rats is able to upregulate L1-CAM expression in the basolateral amygdala and the thalamus (in parallel with behavioral disturbances described above), and secondly they prove that environmental enrichment is able to reverse stress-induced alterations. A final example concerns the role of membrane glycoprotein M6a in stress and neuroplasticity. M6a mRNA was found to be upregulated in the hippocampus of both mice and tree shrews under chronic stress [58], [59]. Cooper and coworkers [43] now establish for the first time that only a splice variant, M6a-Ib, is modulated in a regionally dependent manner, i.e., downregulated in the dentate gyrus granule neurons and in CA3 pyramidal neurons while upregulated in the medial prefrontal cortex.

According to the examples above, it can be concluded that changes in gene expression and their association with behavioral traits or psychopathologies remain among the more powerful experimental tools to uncover the mechanisms leading to a brain disorders. In addition, recent findings demonstrate that complex “epigenetic” mechanisms, which regulate gene activity without altering the DNA code, have long-lasting effects within mature neurons [60]. An example of the former is presented by Collins and coworkers [53], who establish that histone (H3) phospho-acetylation and c-Fos immunoreactivity increase in the dentate gyrus upon exposure to a novel environment or to forced swimming and that their expression is further augmented in exercised rats.

Another fruitful research area in biological psychiatry is the link between neural plasticity, MD, and antidepressants [9], [13], [32], [61]. In particular, neurogenesis in the granular layer of the dentate gyrus is impaired by stress exposure and increased by other environmental factors including environmental enrichment or exercise [9], [61]. Of main interest was the demonstration that hippocampal neurogenesis is required for the beneficial effect of some but not all antidepressant classes [62]–[64]. Although impaired neurogenesis could not be confirmed in a human cohort of MD patients [65], the study of adult hippocampal neurogenesis in MD has benefited tremendously from the attention it has received, and results will ultimately demonstrate its role in the etiology and/or treatment of MD [60]. Oomen and coworkers [46] now demonstrate that maternal deprivation in rats at postnatal day 3, which induces a transient increase of maternal care, also determines impaired neurogenesis in the dentate gyrus (DG) of female rats, while increasing neurogenesis in male rats. Therefore, early environment may have a critical influence on establishing long-held sex differences in neural plasticity. This finding is particularly interesting because MD incidence in women is about twice that for men [66].

## Metabolic Functions

In addition to neuroanatomical changes, MD is also associated with severe vegetative and biological disturbances, including sleep and eating disorders, body weight changes, and neuroendocrine abnormalities. The *DSM-IV* indicates as diagnostic criteria increased or diminished appetite/body weight which should represent a change from pre-MD onset. Most animal models including several discussed in the present *Collection* [30], [43]–[45] describe stress-associated weight loss, which has long being considered a face-validity criterion for a valid animal model of MD [67]. Until recently there was a paucity of animal models of chronic stress-induced weight gain. Bartolomucci and coworkers [42] now report a mouse model of social subordination stress with behavioral depression-like responses and neuroendocrine disturbances, which also determine hyperphagia, weight gain, and increased vulnerability to obesity. In addition, another study in the present *Collection* [46] reports that maternal deprivation determined increased weight gain in juvenile rats when compared with undisturbed controls. These data offer new experimental tools to investigate the link between mood disorders and metabolic functions. In this respect it is remarkable that obesity is often found in comorbidity with MD and particularly so with the atypical depression subtype [68], while clinical efficacy of antidepressants is reduced in obese individuals [69]. Accordingly, there is a great need to rule out the mechanism responsible for stress-induced positive or negative energy balance in different animal models as well as in MD patients.

## “Stress-Induced Depression” (STRID): A New Depression Subtype?

The notion that stress may cause depression has been an underlying concept in the choice of papers included in the *PLoS ONE Collection* discussed here. The link between stress and depression is not novel, and several authors have aimed at identifying new subtypes of depression based on their functional link with stress exposure (e.g., [70]–[72]). Of special interest for this *Collection* is to highlight a potentially common depression subtype, named tentatively “stress-induced depression” (STRID), recently described in Sweden by Åsberg and coworkers [71]. A dramatic increase in the number of workers on long-term sick leave was observed between the years 1997 and 2003 (Statistics Sweden, 2004; http://www.scb.se). Studies of consecutive cases with psychiatric diagnoses culled from the databases of two large Swedish insurance companies showed that about 80% of patients met DSM-IV criteria for MD (Åsberg et al., unpublished data). The depression episodes were mild to moderate (MADRS <20), and accompanied by significant working memory impairment [71]. Follow up showed that STRID tended to have a prolonged course, and that the patients often remained in a state of exhaustion after the depressive symptoms had remitted. Typically, the remaining clinical picture was one of deep mental and physical fatigue, disturbed and non-restorative sleep, irritability, perceptual hypersensitivity, emotional liability, and pronounced cognitive disturbances (mainly memory and concentration problems). A closer examination of the case histories revealed that a majority was clearly induced by psychosocial stress, either at the workplace or often in combination with stress factors in the family. This was confirmed by data obtained in a cohort of almost 5,000 Swedish workers on long-term sick leave with a psychiatric diagnosis [73]. Findings are consistent with the life event stress literature showing that specific, enduring work-related stressful experiences contribute to depression [74]. From an endocrine standpoint, disturbances of the HPA axis may be distinctive pathophysiological features of this depression subtype. HPA-axis hyper-reactivity has long been known and considered a classical feature of depression, particularly with the severe, melancholic type. An opposite situation, i.e., HPA-axis hypo-reactivity was found instead in STRID patients [71]. In addition to the HPA-axis disturbance, the STRID subtype of MD is expected to be linked to different neurobiological, immunological [18], and metabolic features, thus requiring joint forces between preclinical and clinical research.

Overall, the studies presented in this *Special Collection* of *PLoS ONE* propose an integrated effort on how to move in the direction of joint studies. Both preclinical and clinical research will be pivotal in clarifying the validity of this new subtype of MD, in improving predictors for treatment response, and in providing a better basis for genetic studies, as well as in stimulating new drug discovery processes.
